# Levels and Profile of Tetrodotoxins in Spawning *Cephalothrix mokievskii* (Palaeonemertea, Nemertea): Assessing the Potential Toxic Pressure on Marine Ecosystems

**DOI:** 10.3390/toxins17010025

**Published:** 2025-01-06

**Authors:** Grigorii V. Malykin, Peter V. Velansky, Timur Yu. Magarlamov

**Affiliations:** A.V. Zhirmunsky National Scientific Center of Marine Biology, Far Eastern Branch, Russian Academy of Sciences, 690041 Vladivostok, Russia

**Keywords:** tetrodotoxin, TTX, ribbon worm, Nemertea, *Cephalothrix simula*, *Cephalothrix mokievskii*

## Abstract

The ribbon worms of the closely related species *Cephalothrix simula*, *Cephalothrix* cf. *simula*, and *Cephalothrix mokievskii*, representing the *C. simula* species complex, possess high concentrations of tetrodotoxin (TTX) and its analogues in all developmental stages from eggs to adults. It has recently been suggested that the eggs and larvae of these animals can be a source of tetrodotoxins (TTXs) for other aquatic organisms. In the current study, TTXs in mature and post-spawning individuals and in the eggs of *C. mokievskii* were identified using high-performance liquid chromatography–tandem mass spectrometry. For the first time, the quantity and profile of TTXs that these nemerteans released into the environment during spawning were estimated. We showed that the spawning *C. mokievskii* females released significant amounts of TTX and 5,6,11-trideoxyTTX with their eggs; these levels were sufficient for the potential toxification of marine bioresources. The issues surrounding the monitoring of TTXs in commercial marine animals, and collecting at the sites of the spawning of nemerteans from the *C. simula* species complex, are discussed.

## 1. Introduction

Tetrodotoxin (TTX) is a low-molecular-weight guanidinium neurotoxin, which is initially associated with the puffer fishes of the family Tetraodontidae, and has been subsequently discovered in the venom of various marine animals, including blue-ringed octopuses, flatworms, sea slugs, echinoderms, nemerteans, and mollusks [1,2]. In recent years, the contamination of mollusks, especially commercially valuable species, with TTX has attracted increased interest from researchers. Cases of TTX detection in commercial mollusks and the TTX-poisoning incidents following the ingestion of gastropods have been reported worldwide and, increasingly, by European countries [1,3]. Following this trend, the European Food Safety Authority (EFSA) proposed a safety limit of 44 µg/kg TTX in the meat of marine gastropods and bivalves [4]. While the pathway of accumulation of the other group of guanidinium neurotoxins, known as saxitoxins, in mollusks is well known and referred to as algal blooms [5], TTX accumulation is poorly understood. Most studies on TTX migration in marine ecosystems focus on the higher trophic-level species, such as puffer fishes, for which the accumulation of TTX through the food chain is proposed [6,7,8,9]. The exogenous and symbiotic TTX-producing bacteria, that are considered as a primary source of the toxin in marine TTX-bearing animals [10,11,12], have been isolated from several mollusk species [13,14,15] and their habitats [16]. Several studies showed a relationship between the microalgal blooms and TTX occurrence in bivalves, supporting the hypothesis that phytoplankton could be another source of the toxin in mollusks [16,17]. However, trace amounts of TTX, produced by bacteria [11,18], and the absence of direct evidence of TTX synthesis in microalgae, do not allow us to confirm that mollusks accumulate TTX from microorganisms. Since bivalves are filter feeders, the accumulation of TTX during the spawning of other TTX-bearing animals was suggested [17,19,20]. High levels of TTX were detected in the ovaries of many TTX-bearing animals, including puffer fish [21,22,23], flatworms [8,19,24,25], and nemerteans [26]. In a recent study, the ability of bivalves to acquire TTX through feeding on the flatworm larvae was demonstrated in the laboratory [19].

The eggs and larvae of nemerteans from the *Cephalothrix simula* species complex [17,20], which currently includes *C. simula* s. str., *Cephalothrix* cf. *simula*, and *Cephalothrix mokievskii* [27], are considered to be one of the possible sources of TTX in mollusks. An indirect support of the hypothesis of TTX migration from *C. simula* to bivalves was obtained in a recent study by Dhanji-Rapkova and colleagues [20]. Authors detected the DNA of *C. simula* in the digestive tract of the oyster *Crassostrea gigas*. They also found that the concentration of TTX in mollusks reached its highest value during the period of the highest concentration of *C. simula* DNA in seawater. A wide distribution of the nemerteans from the *C. simula* species complex in the Pacific region, along with their frequent detection in the habitats of wild and mariculture oyster farms [28,29,30], support the above hypothesis. Since the early 1990s, the habitat of *C. simula* was considered to be limited to the Sea of Japan [28,31]. However, further reports have provided evidence for the introduction of *C. simula* to the other regions of the world, including the coasts of Portugal, Spain, Italy [30,32], the Netherlands [33], England [34], and the USA [35]. The other members of the *C. simula* species complex, *C*. cf. *simula* and *C. mokievskii*, are found in the Sea of Japan and in the Sea of Okhotsk [36].

In this study, we investigated the content of TTX and its analogues in the nemertean *C. mokievskii* during spawning. A previous study showed that in a small sampling of *C. mokievskii* of an unknown sex, five out of ten animals contained high concentrations of tetrodotoxins (TTXs) [27]. The levels of TTXs found in *C. mokievskii* correspond to those in strongly toxic animals, as defined by Noguchi and Arakawa [6]. The current investigation includes eighty-eight individual *C. mokievskii*, collected along the coasts of Sakhalin Island (Sea of Okhotsk), where this species is abundant. Using high-performance liquid chromatography with tandem mass spectrometry (HPLC-MS/MS), we determined the concentration and profile of TTXs in mature and post-spawning animals and eggs. The issues of a potential toxic load on the ecosystem and on mollusk communities during the spawning of *C. mokievskii*, and the subsequent potential risk to human health, are addressed.

## 2. Results

**Figure 1:**
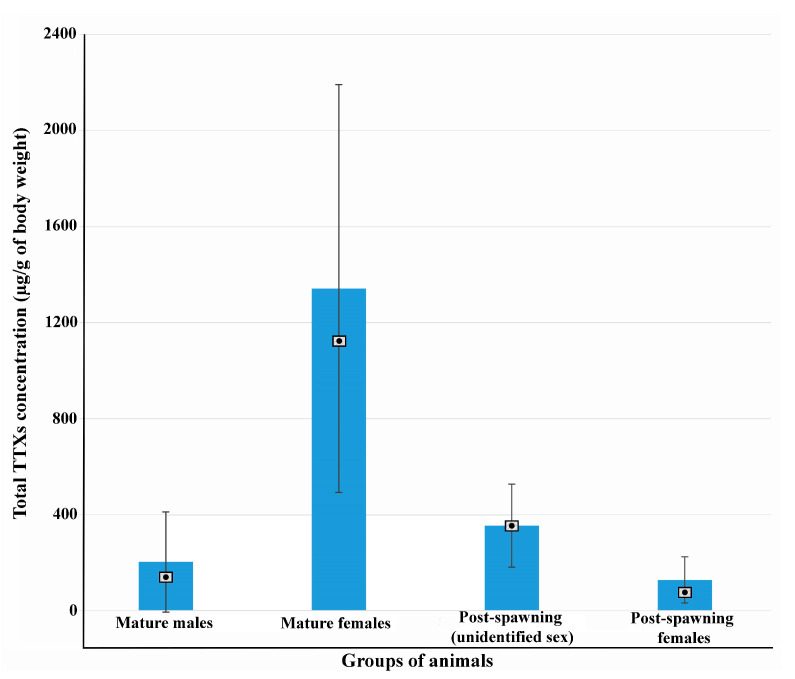
The mean total concentrations of tetrodotoxin and its analogues (TTXs) in mature males, mature females, post-spawning individuals of unidentified sex, and post-spawning *Cephalothrix mokievskii* females collected in the Aniva Bay, Sea of Okhotsk. Rectangles with dots show median values.

The differences in TTX concentrations between mature males, mature females, post-spawning individuals of unidentified sex, and post-spawning *C. mokievskii* females were evaluated using the Kruskal–Wallis H test. The test showed that TTX concentrations in the studied groups were statistically different (*p*-value < 0.05). Pairwise comparison of studied groups using the Mann–Whitney U test revealed that TTX concentrations were statistically different between all groups (*p*-value > 0.05), except mature males and post-spawning females. TTX concentrations between mature males and post-spawning females did not differ significantly (*p*-value < 0.05). Figure 1 shows the mean values of total TTX concentrations for the studied groups with a standard deviation and median values.

**Figure 2:**
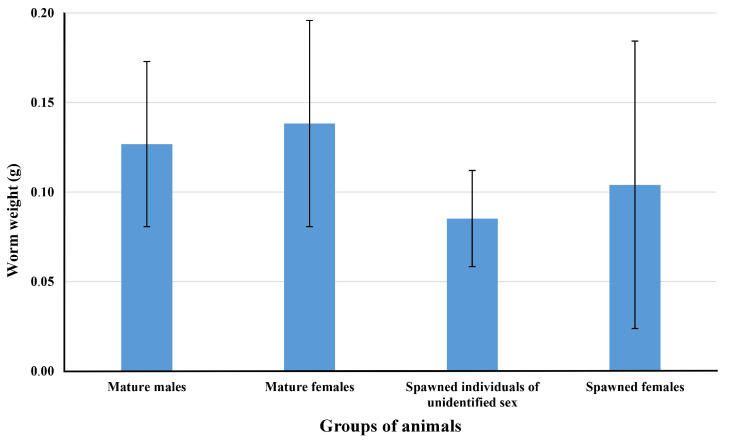
Average weight of *Cephalothrix mokievskii* individuals from Aniva Bay, Sea of Okhotsk.

The differences in weight between mature males, mature females, post-spawning individuals of unidentified sex, and post-spawning *C. mokievskii* females were evaluated using the Kruskal–Wallis H test. The test showed that the weights of individuals in the studied groups were not statistically different (*p*-value > 0.05). Figure 2 shows the mean values of individual masses for the studied groups, with a standard deviation.

**Figure 3:**
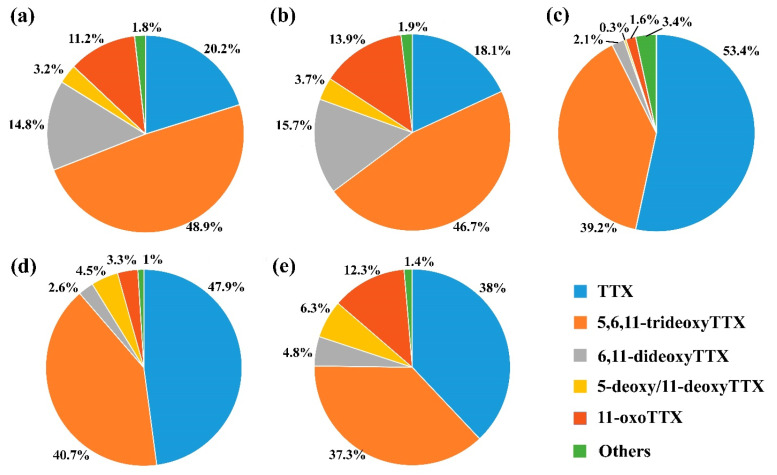
The percent proportions of the mean concentrations of tetrodotoxin and its analogues in the *Cephalothrix mokievskii* collected in Aniva Bay, Sea of Okhotsk. (**a**) Mature males. (**b**) Post-spawning individuals of unidentified sex. (**c**) Eggs. (**d**) Mature females. (**e**) Post-spawning females.

In the bodies of all mature and post-spawning animals, between seven and nine TTXs, specifically TTX, 4-epiTTX, 5,6,11-trideoxyTTX, 6,11-dideoxyTTX, 4,9-anhydroTTX, 5-deoxy/11-deoxyTTX, 11-norTTX-6(S/R)-ol, 4-epi-11-oxoTTX, and 11-oxoTTX, were detected (Supplementary Material Table S1 and Figure S1). The spectrum of TTXs in the bodies of mature males and post-spawning individuals of unidentified sex was similar (Figure 3).

The total concentration of TTXs in mature nemerteans ranged from 54 µg/g to 3087.4 µg/g of the total body weight. The mean total concentration of TTXs in females exceeded that in males by approximately 6.5-fold (Figure 1, Supplementary Material Table S1).

In post-spawning nemerteans, the total concentrations of TTXs ranged from 35.7 µg/g to 864.1 µg/g of the total body weight. The mean total concentrations of TTXs in post-spawning females and individuals of unidentified sex was lower than in mature females by approximately 10.5- and 3.8-fold, respectively (Figure 1, Supplementary Material Table S1).

The samples of eggs contained between five and seven TTXs, specifically TTX, 4-epiTTX, 5,6,11-trideoxyTTX, 6,11-dideoxyTTX, 4,9-anhydroTTX, 5-deoxy/11-deoxyTTX, and 11-oxoTTX (Supplementary Material Table S2). The total amount of TTXs per egg varied from 3.4 ng to 6.4 ng (Supplementary Material Table S2).

The toxins, comprising at least 5% of the total concentration of TTXs per sample, were counted as major. The toxins, comprising less than 5% of the total concentration of TTXs per sample, were counted as minor. Mature females contained between two and five major toxins (Supplementary Material Table S1). TTX (31.4–61%) and 5,6,11-trideoxyTTX (30.3–55.7%) were presented as major in all females (Figure 3d). The number of minor toxins in mature females varied from four to seven, and their total sum ranged from 2.9% to 12.9% (Supplementary Material Table S1). Mature males contained between four and five major toxins (Supplementary Material Table S1). TTX (5.6–38%), 5,6,11-trideoxyTTX (27.6–75%), 6,11-dideoxyTTX (5.9–25.2%), and 11-oxoTTX (5–22.1%) were counted as major in most mature males (Figure 3a). The number of minor toxins in mature males varied from four to five, and their total sum ranged from 1.6 to 7.1% (Supplementary Material Table S1). Post-spawning nemerteans contained between three and five major toxins (Supplementary Material Table S1). TTX (10.4–35.2%), 5,6,11-trideoxyTTX (21–63.5%), 6,11-dideoxyTTX (5.2–42.6%), and 11-oxoTTX (6.4–22.4%) were counted as major in most post-spawning individuals of unidentified sex (Figure 3b). TTX (22–57.7%), 5,6,11-trideoxyTTX (22.9–49%), and 11-oxoTTX (6.8–20.2%) were counted as major in all post-spawning females (Figure 3e). The number of minor toxins in post-spawning nemerteans varied from four to six, and their total sum ranged from 0.9 to 9% (Supplementary Material Table S1). Eggs contained two major toxins–TTX (50.8–57.3%) and 5,6,11-trideoxyTTX (34.7–43.6%) (Figure 3c). The number of minor toxins in the eggs was six, and their total sum ranged from 3.3 to 9.8% (Supplementary Material Table S2).

## 3. Discussion

In subsequent years, the presence of TTXs in nemerteans of the *C. simula* species complex was reported by various scientific groups. And although the concentration between individuals of different species and between individuals collected in different locations may vary significantly, representatives of this species complex can contain significant amounts of TTXs, comparable with the amounts found in blue-ringed octopuses and puffer fishes. The amount of TTXs in *C. simula* individuals collected from the intertidal zone in Shimoda (Shizuoka prefecture, Japan) and in the Hiroshima Bay (Japan) reached up to 23,000 MU/g (4094 μg/g) [30] and 25,590 MU/g (4555 μg/g) [28] of the body weight, respectively According to Vlasenko and Magarlamov [37], TTX concentrations in individual *C*. cf. *simula*, collected from the Peter the Great Bay (See of Japan), varied from 85.75 μg/g to 7108.26 μg/g. An individual *C. simula* collected off of the coast of England had a total TTX concentration of 54.3 μg/g [33]. In the current study, we have shown that individual *C. mokievskii* contained between 54 µg/g and 3087.4 µg/g of TTXs (Supplementary Material Table S3). Thus, in the current study, we have shown that the concentration of TTX in *C. mokievskii* can also vary significantly (even in individuals at the same maturation stage (Figure 1)), but the reasons for this have yet to be clarified.

The nemertean *C. simula* from the *C. simula* species complex is one candidate for TTX transfer to bivalves [17,20]. In the present study, we, for the first time, showed that mature *C. mokievskii* females (another member from the *C. simula* species complex) contain TTXs (50% of which comprised TTX) in their eggs, but not in other tissues. We assume that the TTX-containing eggs of *C. mokievskii,* as well as larvae hatched from them, can toxify bivalves in the breeding sites of this species, distributed in the Sea of Japan and the Sea of Okhotsk.

Despite the increasing interest in the hypothesis of TTX migration from nemerteans to bivalves, it is not yet clear how much toxins the worms release during spawning and what toxic load on mollusks or the ecosystem can be expected. Our results show that the mature individual *C. mokievskii* contain significant amounts of TTXs (up to 3087.4 µg/g), with females containing more toxins than males by approximately 1–2 orders of magnitude (Figure 1). Moreover, the level of TTXs in individuals before and after spawning differs mainly in females. The level of TTXs in females after spawning can be 100-fold lower compared to the females before spawning, while the weight of spawned and mature animals is not statistically different (Figure 2). Although in *C. mokievskii* males, reproductive products can contribute to the toxification of bivalves, eggs can be considered as the main source of toxins for these animals. According to the obtained data, a female *C. mokievskii* can lose up to 420 µg of TTXs during spawning. In theory, for a 3-year-old mussel weighting 150 g, an intake of 6.7 μg of TTXs, corresponding to the toxins content in approximately 1000 oocytes of *C. mokievskii*, would be sufficient to exceed the recommended limit of TTX (44 μg/kg) set by EFSA [4]. Thus, the amount of TTXs, which one *C. mokievskii* female releases with eggs during spawning, is sufficient to toxify about 60 mollusks. The attention to the monitoring of the level of TTXs in bivalves during the spawning period of TTX-bearing nemerteans seems reasonable.

Since different TTXs could affect human health differently, attention should be paid to the spectrum of toxins which the bivalves accumulated. In the extracts of mature individual *C. mokievskii*, up to nine TTXs were detected, while in the eggs the maximum of seven toxins were observed. More than 90% of the TTX amount in the eggs was composed of TTX (about 50%) and 5,6,11-trideoxyTTX (about 40%). Similar patterns in the distribution of TTXs can be traced for the other aquatic organisms, which contain a wide range of toxins in the body and eggs, while TTX and 5,6,11-trideoxyTTX remain the predominant toxins in the eggs. This was demonstrated for the nemertean *C*. cf. *simula*, collected in the West coast of the Sea of Japan [26], flatworm *P. multitentaculata*, collected in the coastal area of Hayama, Kanagawa (Japan) [8], and puffer fish *Lagocephalus sceleratus*, collected from the South Crete coast [38]. It is unclear whether the eggs accumulate predominantly TTX and 5,6,11-trideoxyTTX due to the peculiarities of the intrabody TTX transport, or the physicochemical properties of these toxins. However, it seems that the profile of TTXs in the eggs and larvae of the animals, presumably involved in bivalve toxification, do not depend on their species or geographical location. Although the majority of studies of TTXs in mollusks revealed only TTX, in a case study on shellfish poisoning in the Mediterranean Sea in 2008, poisoned mollusks contained high concentrations of both TTX and 5,6,11-trideoxyTTX [39]. Despite the fact that, among the major TTXs found in eggs, TTX is the most dangerous for humans with a half-maximal inhibitory concentration (IC_50_) of 5.43 nM [40], 5,6,11-trideoxyTTX with IC_50_ of 4196 nM [40] can also pose a threat, acting like a TTX agonist, and enhancing the toxic effect [40].

## 4. Conclusions

This study shows, for the first time, that the female nemerteans of *C. mokievskii* release significant amounts of TTX and 5,6,11-trideoxyTTX during spawning and can be a potential source of these toxins in marine ecosystems. The origin of TTX in highly toxic animals, including nemerteans from the *C. simula* species complex, remains unclear. One hypothesis states that TTX can be produced by free-living and/or symbiotic microflora and transmitted through the food web, accumulating in high concentrations in animals at higher trophic levels. While it is impossible to define the reason for higher TTX levels in female *C. mokievskii* at the moment, they can be considered as a promising model for the study on the origin of TTXs in nemerteans. The present study also opens up perspectives regarding the investigating of TTX transfer from the eggs of nemerteans from the *C. simula* species complex to commercial bivalves in the artificial toxification experiments.

## 5. Materials and Methods

**Figure 4:**
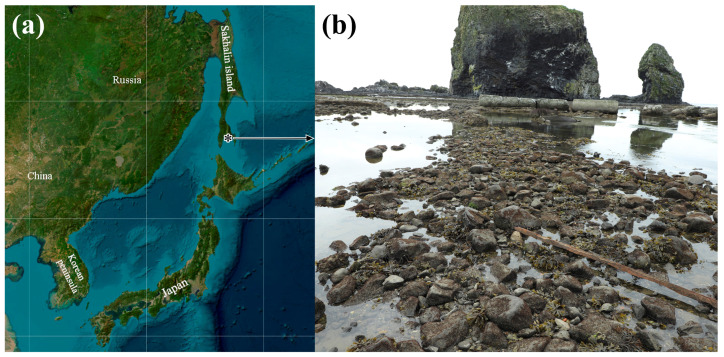
The locality of *Cephalothrix mokievskii* sampling. (**a**) Geographical location of the sampling area (asterisk); (**b**) the habitat of *C. mokievskii*.

In total, 88 samples of *C. mokievskii* (26 mature males, 24 mature females, 25 post-spawning individuals of unidentified sex, and 13 post-spawning females) were collected from their habitat from the bottom face of stones in the upper littoral of Aniva Bay, Sea of Okhotsk (46.02° N, 142.17° E), at 15–17 August 2024 by Timur Yu. Magarlamov (Figure 4). The biological material was collected from the not-protected area, not requiring a Research Access or Field Permit. Manipulations with animals were performed according to ARRIVE guidelines (https://arriveguidelines.org/arrive-guidelines, accessed on 14 July 2020).

Prior to the experiments, the animals were stored separately in the tanks with aerated, filtered seawater at 17 °C for 1–3 h. To filter the seawater, 0.45 µm pore membrane (hydrophobic polyvinylidene fluoride (PVDF)) filters were used (Merck Millipore, Burlington, MA, USA). Individuals were washed three times before fixation in filtered seawater.

**Figure 5:**
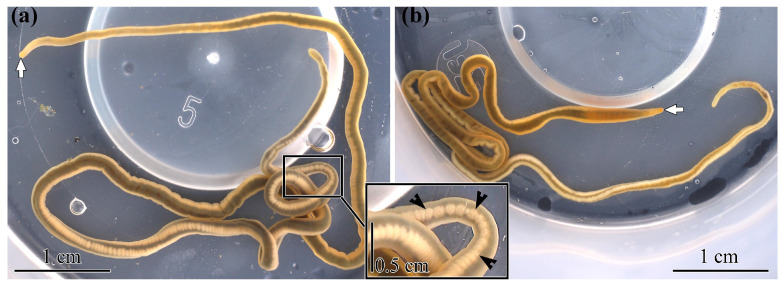
Live specimens of *Cephalothrix mokievskii*, collected on 15 August 2024 in Aniva Bay, Sea of Okhotsk. Arrows indicate the head. (**a**) Female. Inset shows the body with eggs (arrowheads). (**b**) Male.

A preliminary identification of *C. mokievskii* was performed based on morphological features. The species is characterized by a thin body, which can reach a length of up to 30 cm and a width of up to 1–3 mm. The body of this worm has a dark yellow coloration, often with a greenish tinge. The tip of the head is pointed, and often has a bright orange color. *C. mokievskii* has dark intestine, which Korotkevich [41] described as a “dark mediodorsal stripe” (Figure 5).

For the genetic identification of specimens, they were fixed in 96% ethanol. Genomic DNA was extracted from specimens using DNAZol (Thermo Fisher Scientific, Waltham, MA, USA) according to the protocol recommended by the manufacturer. A fragment of the mitochondrial cytochrome *c* oxidase subunit I (COI) gene was amplified using the LoboF1 (5′-KBTCHACAAAYCAYAARGAYATHGG-3′) and LoboR1 (5′-TAAACYTCWGGRTGWCCRAARAAYCA-3′) primers (Lobo et al., 2013). The amplification was performed with the following conditions: denaturation at 94 °C for 5 min, followed by 5 cycles at 94 °C for 30 s, 45 °C for 90 s, and 72 °C for 60 s, followed by 45 cycles at 94 °C for 30 s, 54 °C for 90 s, 72 °C for 60 s, and a final elongation at 72 °C for 5 min. The amplified products were purified using an QIAquick PCR Purification Kit (Qiagen, Hilden, Germany). The direct sequencing of both DNA strands was carried out using the BigDye Terminator ver. 3.1 Cycle Sequencing Kit (Applied Biosystems, Waltham, MA, USA) and the same PCR primers using an ABI Prism 3500 Genetic Analyzer (Applied Biosystems, Waltham, MA, USA). BLAST searches were performed as implemented in the NCBI website (http://www.ncbi.nlm.nih.gov/, accessed on 9 December 2024). The resulting sequences were submitted to the DDBJ/ENA/GenBank databases (PQ724485).

Spawning was induced by a rapid temperature increase. Mature female individuals were placed in a separate Petri dish with filtered seawater at 27 °C. Eggs were collected in a sterile 2 mL tube with 96% ethanol.

For TTX extraction, the specimens of adult nemerteans (one specimen = the total extract of one individual) and eggs (one specimen = the total extract of 10 eggs) were fixed in 2 mL of 96% ethanol. The specimens of adult worms were additionally homogenized in a 0.1% solution of acetic acid in 70% ethanol (with a specimen/solution ratio of 1:10 *v*/*v*) for 5 min using a hand-held homogenizer. After that, all the specimens were ultrasonicated on a Sonopuls HD 2070 homogenizer (Bandelin, Berlin, Germany) for 5 min (at a frequency of 20 kHz; amplitude, 228 µm; working cycle, 0.8 s; and interval, 0.2 s) and were centrifuged at 14,000× *g*, 10 min, 4 °C; then, the supernatants were collected. The obtained sediments were extracted twice, using a 0.1% solution of acetic acid in 70% methanol (with a specimen/solution ratio of 1:1 *v*/*v*); after centrifugation, the supernatants were pooled. The specimens were dried on a rotary evaporator (Labconco, Kansas City, MO, USA) at 60 °C. The dry precipitates were dissolved in a 0.1% aqueous solution of acetic acid and concentrated using 3 kDa Vivaspin turbo concentrators (Sartorius, Goettingen, Germany).

TTXs were identified and quantified using HPLC-MS/MS according to the methodology Bane et al. [42] with modifications according to the methods of Malykin et al. [26] (Supplementary Material File S1). The limit of quantification (LoQ) was 0.6 ng/mL (0.0006 µg/g); the limit of detection (LoD) was 0.2 ng/mL (0.0002 µg/g).

Statistical analyses were conducted using the Statistica toolbox version 10.0 (Dell, Round Rock, TX, USA). TTXs values between the groups of specimens (mature males, mature females, post-spawning individuals of unidentified sex, and post-spawning females) were compared using the Kruskal–Wallis H test and the Mann–Whitney U test (*p*-value < 0.05 was considered statistically significant).

## Data Availability

The original contributions presented in this study are included in the article/supplementary material. Further inquiries can be directed to the corresponding author.

## Supplementary Materials

The following supporting information can be downloaded at: http://www.mdpi.com/article/10.3390/toxins17010025/s1. Supplementary Table S1: Tetrodotoxins concentrations of mature animals of *Cephalothrix mokievskii*. Supplementary Figure S1: Chromatograms and spectra of putative tetrodotoxins. Supplementary Table S2: Tetrodotoxins concentration in eggs of *Cephalothrix mokievskii*. Supplementary Table S3: Tetrodotoxins initial data. Supplementary File S1: Parameters of tetrodotoxins analysis, according to [43,44,45,46,47,48].
