# Supplementary material for: Levels and Profile of Tetrodotoxins in Spawning Cephalothrix mokievskii (Palaeonemertea, Nemertea): Assessing the Potential Toxic Pressure on Marine Ecosystems
Source: Toxins (Basel). 2025 Jan 6;17(1):25. doi: 10.3390/toxins17010025 (PMC11768988; doi:10.3390/toxins17010025)
Supplement: Supplementary file 1 [file toxins-17-00025-s001.zip › Figures S1 and S2.pdf]

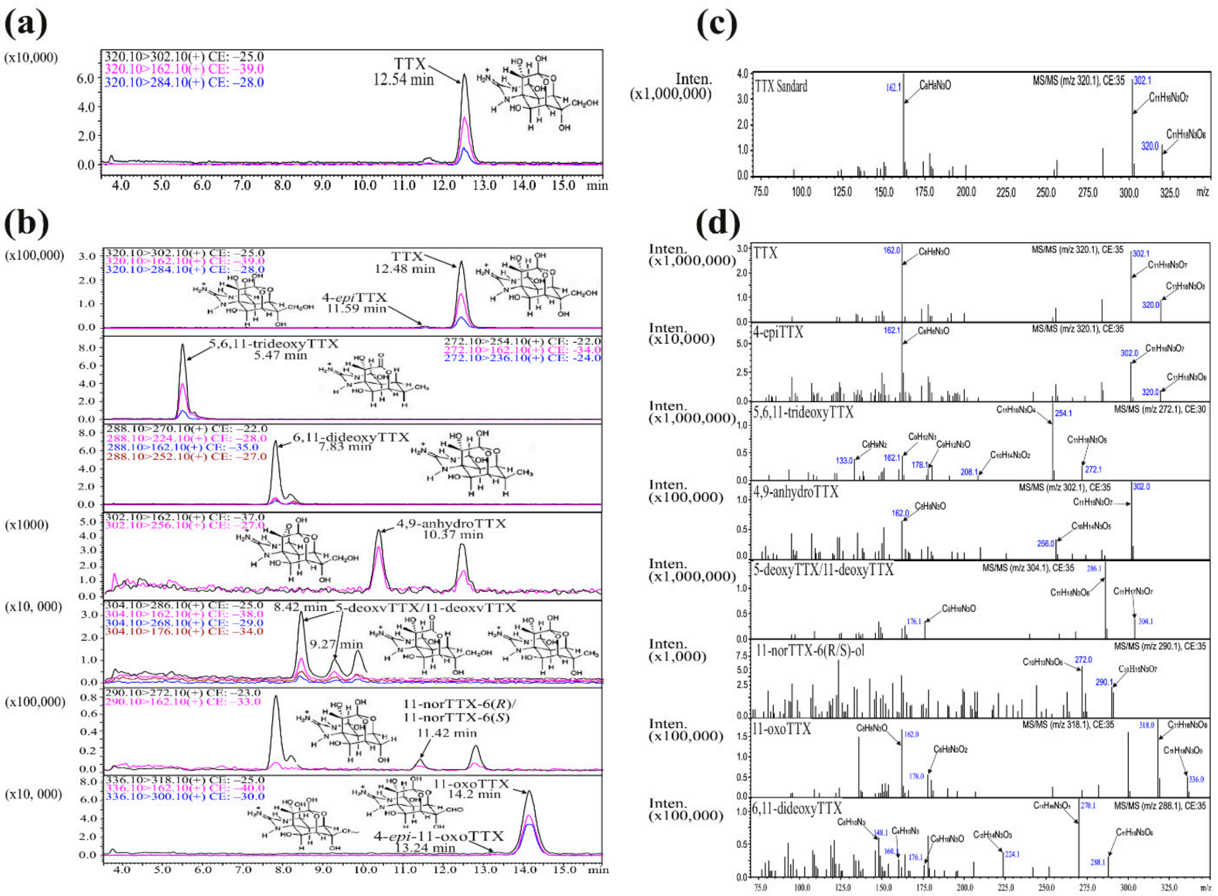

Figure S1. Chromatograms (**a**,**b**) and spectra (**c**,**d**) of tetrodotoxin (TTX) and its analogues obtained by high-performance liquid chromatography–tandem mass spectrometry (HPLC–MS/MS). (**a**) Chromatogram of TTX standard; (**b**) Chromatograms of TTX and its analogues from extracts of *Cephalothrix mokievskii*. The curves of different colors are for different mass transitions (described in each chromatogram); (**c**) Spectrum of TTX standard; (**d**) Spectra of TTX and its analogues from extracts of *Cephalothrix mokievskii*.

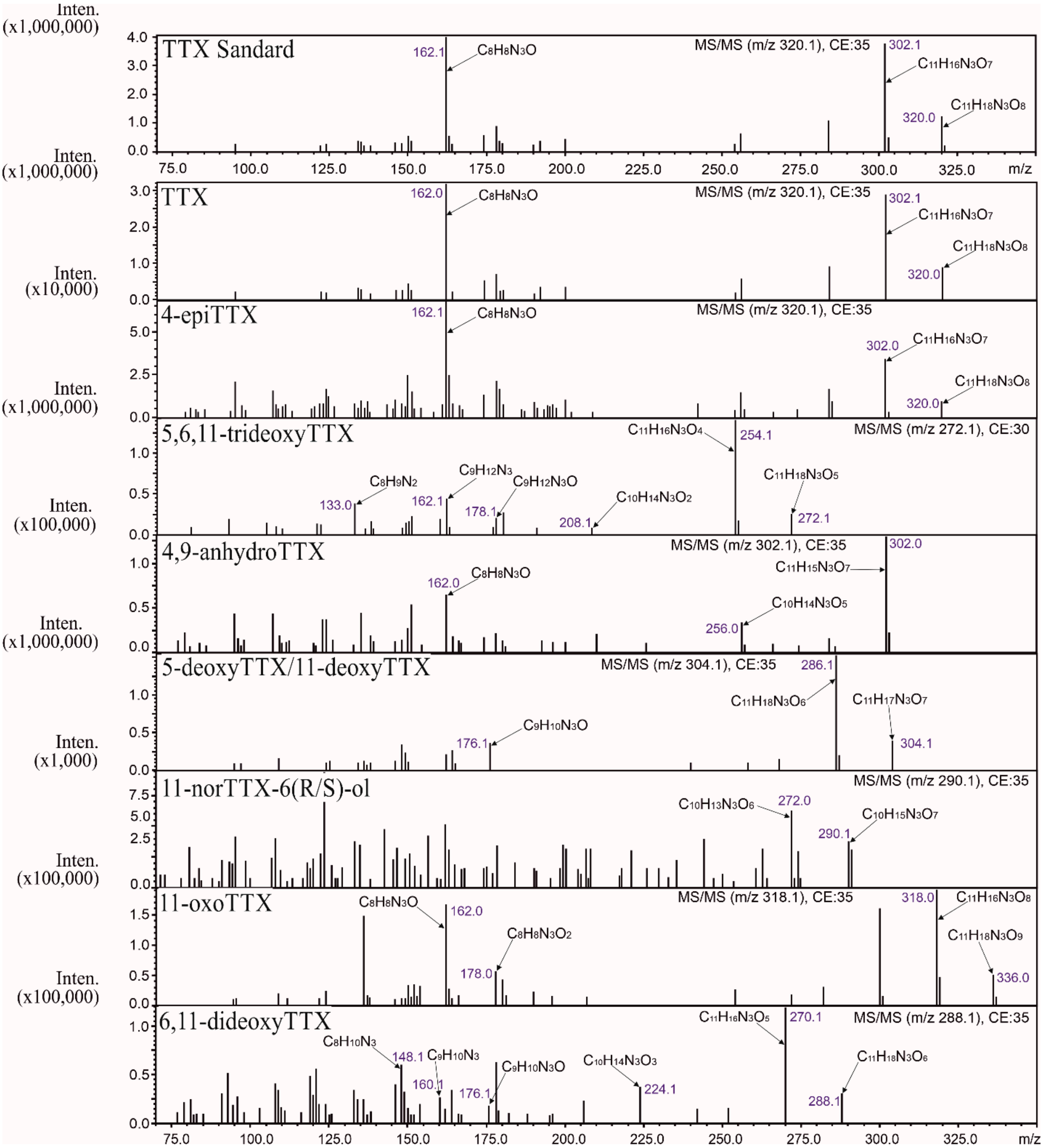

Figure S2. High-performance liquid chromatography with tandem mass spectrometry (HPLCMS/MS) spectra of tetrodotoxin (TTX) standard and TTX and its analogues from extracts of *Cephalothrix mokievskii*.
