# Supplementary material for: Levels and Profile of Tetrodotoxins in Spawning Cephalothrix mokievskii (Palaeonemertea, Nemertea): Assessing the Potential Toxic Pressure on Marine Ecosystems
Source: Toxins (Basel). 2025 Jan 6;17(1):25. doi: 10.3390/toxins17010025 (PMC11768988; doi:10.3390/toxins17010025)
Supplement: Supplementary file 1 [file toxins-17-00025-s001.zip › File S1 HPLC-MS_MS Parameters.pdf]

Parameters of tetrodotoxin (TTX) and its analogues analysis

Tetrodotoxin and its analogues (TTXs) were identified using HPLC–MS/MS. The HPLC system included two pairs of LC-30 pumps, a SIL-30AC autosampler, a CTO-20A thermostat, an SCL-20A system controller, and a triple quadrupole mass spectrometer LCMS-8060 (Shimadzu Europa, Duisburg, Germany) with electrostatic spray ionization (ESI). Separation was carried out using a SeQuant ZIC HILIC column (150 × 2.1 mm, 5 μm) (Merck, Darmstadt, Germany) at 40 °C and a flow rate of 0.25 ml/min. A binary gradient was used: mobile phase A, ammonia (5 mM) and formic acid (8 mM) in 9:1 acetonitrile–water; mobile phase B, ammonia (10 mM) and formic acid (20 mM) in water. A gradient profile was used as follows: (a) 0–4.3 min, 15% B; (b) 4.4–17 min, 25% B; (c) 17–20 min, 50% B. The sample volume was 1 μL. A SeQuant ZIC-HILIC guard column (20 × 2.1 mm, 5 μm) (Merck, Darmstadt, Germany) was installed in line before the analytical column through a two-position 6-port valve. At 4.4 min, the valve was switched and guard columns backflashed with isopropanol (4.4–7 min) and water (7–14 min) at flow rate of 0.2 ml/min. At 17 min, the valve was switched back. The mass spectrometer was operated in scan (m/z 200–1000) and multiple reaction monitoring (MRM) modes. The ion source parameters were as follows: interface temperature—280°C, desolvation line temperature—250°C, nebulizing gas (N2) flow—3 L/min, drying gas (N2) flow—3 L/min, and heating gas (dry air) flow—17 L/min. The TTX concentration was calculated using the calibration curve of a standard TTX (purity > 99%) solution series (Cat No: T-550, Alomone Labs Ltd., Jerusalem, Israel). The toxin detection criteria included a precursor MRM transition peak S/N ratio > 3, relative intensity of the fragment ion peak > 4%. MRM transitions, collision energy and the order of toxins elution (retention time) were used to detect TTX analogues as described by Bane et al. (2014)[42], Shoji et al. (2001)[43], Vale (2008)[44], Kudo et al. (2012)[45], Yotsu-Yamashita et al. (2013)[46], and Turner et al. (2017)[47] and indicated in Table 1. The concentrations of TTX analogues were calculated following the procedure of Chen et al. (2011)[48]. The method was validated using standard TTX solutions in MRM mode. The linearity range was from 0.0006 to 0.1 μg/g. The recovery range from 0.001 to 0.1 μg/g of TTX was 98.4%. The limit of quantification (LoQ) was 0.0006 μg/g (0.6 ng/mL). The limit of detection (LoD) was 0.0002 μg/g (0.2 ng/mL), and the relative SD was 4.5%–14.6%.

Table 1 MRM transitions, collision energy and retention time for TTX and its analogues.

**№**	**Analogue**	**MRM transitions**	**Collision Energy (eV)**	**Retention time (min)**
1	TTX	320.1>302.1<br>320.1>162.1	25, 38<br>36.949–55.423	12.48

2	4-epiTTX	320.1>302.1<br>320.1>162.1	25, 38	11.51
3	5,6,11-trideoxyTTX	272.1>162.1<br>272.1>254.1<br>208.1064;<br>180.1137;<br>178.0954;<br>162.1020;<br>133.0724;	33.570–50.358	5.64
4	4,9-anhydroTTX	302.1>256.1<br>302.1>162.1	30<br>49	10.32
5	11-oxoTTX	336>318<br>336>300<br>336>282<br>336>178<br>336>162	38.1–57.1	13.9
6	4-epi-11-oxoTTX	320.1>302.1<br>320.1>162.1	25–40	13.37
7	5-deoxyTTX/11-deoxyTTX*	304>286<br>304>176	49	9.8<br>9.21
8	11-norTTX-6(R)-ol /11-norTTX-6(S)-ol*	290/272<br>290.1>162.1	49<br>30	10.33, 11.39
9	6,11-dideoxyTTX	288>224	34.6831–52.046	8.18

* toxins are not differentiated.

References

42. Bane, V.; Brosnan, B.; Barnes, P.; Lehane, M.; Furey, A. High-Resolution Mass Spectrometry Analysis of Tetrodotoxin (TTX) and Its Analogues in Puffer Fish and Shellfish. *Food Addit. Contam. Part A* **2016**, *33*, 1468–1489. https://doi.org/10.1080/19440049.2016.1218070.

43 Shoji Y, Yotsu-Yamashita M, Miyazawa T, Yasumoto T (2001) Electrospray ionization mass spectrometry of tetrodotoxin and its analogs: liquid chromatography/mass spectrometry, tandem mass spectrometry, and liquid chromatography/tandem mass spectrometry. Anal Biochem 290:10–17. https://doi.org/10.1006/abio.2000.4953

44 Vale P (2008) Complex profiles of hydrophobic paralytic shellfish poisoning compounds in Gymnodinium catenatum identified by liquid chromatography with fluorescence detection and mass spectrometry. J Chromatogr A 1195:85–93. https://doi.org/10.1016/j.chroma.2008.04.073

45 Kudo Y, Yasumoto T, Konoki K, et al (2012) Isolation and structural determination of the first 8-epi-type tetrodotoxin analogs from the newt, Cynops ensicauda popei, and comparison of tetrodotoxin analogs profiles of this newt and the puffer fish, Fugu poecilonotus. Mar Drugs 10:655–667. https://doi.org/10.3390/md10030655

46 Yotsu-Yamashita M, Abe Y, Kudo Y, et al (2013) First identification of 5,11-dideoxytetrodotoxin in marine animals, and characterization of major fragment ions of tetrodotoxin and its analogs by high resolution ESI-MS/MS. Mar Drugs 11:2799–2813. https://doi.org/10.3390/md11082799

47 Turner AD, Dhanji-Rapkova M, Coates L, et al (2017) Detection of tetrodotoxin shellfish poisoning (TSP) toxins and causative factors in bivalve molluscs from the UK. Mar Drugs 15:1–18. https://doi.org/10.3390/md15090277

48 Chen XW, Liu HX, Jin YB, et al (2011) Separation, identification and quantification of tetrodotoxin and its analogs by LC-MS without calibration of individual analogs. Toxicon 57:938–943. https://doi.org/10.1016/j.toxicon.2011.03.011
